# Appropriate Osteoporosis Treatment by Family Physicians in Response to FRAX vs CAROC Reporting: Results From a Randomized Controlled Trial

**DOI:** 10.1016/j.jocd.2013.09.007

**Published:** 2013-10-25

**Authors:** Karen A. Beattie, George Ioannidis, Joy C. MacDermid, Ruby Grewal, Alexandra Papaioannou, Jonathan D. Adachi, Anthony B. Hodsman

**Affiliations:** 1 Department of Medicine, McMaster University, Hamilton, ON, Canada; 2 School of Rehabilitation Science, McMaster University, Hamilton, ON, Canada; 3 Department of Surgery, Western University, St. Joseph’s Health Centre, London, ON, Canada; 4 Department of Medicine, McMaster University, St. Peter’s Hospital, Hamilton, ON, Canada; 5 Department of Medicine, Western University, St. Joseph’s Health Centre, London, ON, Canada

**Keywords:** Bone mineral density, decision making, fracture risk, FRAX, osteoporosis, treatment recommendation

## Abstract

Canadian guidelines recommend either the FRAX or the Canadian Association of Radiologists and Osteoporosis Canada (CAROC) fracture risk assessment tools to report 10-yr fracture risk as low (<10%), moderate (10%–20%) or high (>20%). It is unknown whether one reporting system is more effective in helping family physicians (FPs) identify individuals who require treatment. Individuals ≥50 yr old with a distal radius fracture and no previous osteoporosis diagnosis or treatment were recruited. Participants underwent a dual-energy x-ray absorptiometry scan and answered questions about fracture risk factors. Participants’ FPs were randomized to receive either a FRAX report or the standard CAROC-derived bone mineral density report currently used by the institution. Only the FRAX report included statements regarding treatment recommendations. Within 3 mo, all participants were asked about follow-up care by their FP, and treatment recommendations were compared with an osteoporosis specialist. Sixty participants were enrolled (31 to FRAX and 29 to CAROC). Kappa statistics of agreement in treatment recommendation were 0.64 for FRAX and 0.32 for bone mineral density. The FRAX report was preferred by FPs and resulted in better postfracture follow-up and treatment that agreed more closely with a specialist. Either the clear statement of fracture risk or the specific statement of treatment recommendations on the FRAX report may have supported FPs to make better treatment decisions.

## Introduction

Despite guidelines for diagnosing and managing osteoporosis (1–3), few individuals who experience a fragility fracture are evaluated for osteoporosis, and even fewer are treated (4–6). This is concerning because experiencing a fracture after age 50 yr is a major risk factor for future fractures (7–9) and medications reduce the risk of fractures by 40% –70% (10–12). A significant care gap exists between the number of individuals who should be treated for osteoporosis and those who receive treatment (5,6,13–15), reinforcing the need for the application of clinical guidelines (16,17). Barriers to the adaptation of guidelines include a lack of knowledge about risk factors and the interpretation of bone mineral density (BMD) results (18–22).

The World Health Organization’s fracture risk assessment tool (FRAX) (23) and the Canadian Association of Radiologists and Osteoporosis Canada (CAROC) (3) fracture risk assessment tool improve the identification of patients who would benefit from treatment by categorizing them as having a low (<10%), moderate (10%–20%), or high (>20%) fracture risk over 10 yr. These tools, both of which are used in Canada (1), integrate clinical risk factors for fracture with BMD. Accordingly, they improve the sensitivity and specificity of fracture prediction (24–26). Clinical risk factors included in FRAX are age, previous fragility fracture, parental history of hip fracture, smoking status, high alcohol intake, systemic use of corticosteroids, low body mass index, and diseases associated with secondary osteoporosis (26–29). The CAROC tool includes sex, age, and femoral neck BMD with prevalent fragility fractures and corticosteroid incorporated as step-wise categorical modifiers of baseline fracture risk. The FRAX tool estimates precise risk of a major osteoporotic fracture (in percent), whereas the CAROC tool confers only categorical risk.

Controlled trials have assessed whether changes in knowledge translation about osteoporosis guidelines and fracture risk factors result in more appropriate treatment (14,30,31). In one study, authors recommended future studies improve the treatment rate by using ‘‘more clinically useful and directive density reports’’ (14). Another study suggested that, in terms of clinical decision-making, the structure of BMD reporting may be as important as the accuracy of BMD measurements (32).

We attempted to close the care gap by testing whether the implementation of a FRAX report would assist family physicians (FPs) in identifying patients who would benefit from treatment. Compared with a standard CAROC-generated BMD report, we hypothesized that a FRAX report would better convey fracture risk to FPs thereby improving the treatment rate of patients at high fracture risk.

## Materials and Methods

Individuals ≥50 years treated at the Hand and Upper Limb Center (HULC, London, ON) within 1 month of sustaining a fragility fracture of the distal radius were enrolled. Individuals were excluded if they (1) had previously been diagnosed with osteoporosis, (2) were currently taking osteoporosis medications, (3) did not have an FP, or (4) were unable/unwilling to provide informed consent. Participants completed the Self-Administered Comorbidity Questionnaire (33) and the International Osteoporosis Foundation Risk Test (34) before undergoing bone densitometry (GE Lunar Prodigy Advance system, GE Healthcare, Madison, WI). Participants’ FPs were randomly allocated to receive either a standard CAROC-based BMD report used at this center (hereafter referred to as the standard BMD report) or a customized report based on the FRAX model (hereafter referred to as the FRAX report). All reports were generated by a single physician (A.B.H.), who was not blinded to group allocation. Group allocation was concealed to the remaining study investigators. FPs were not aware their patients were participating, and neither participants nor FPs were aware of the study’s primary outcome. Individuals whose FP had already been entered into the study through a previous participant were excluded.

Standard BMD reports (Appendix 1) included participants’ femoral neck and lumbar spine BMDs and T-scores. Fracture risk category was determined with the CAROC tool; the wrist fracture increased the baseline risk category into the next highest category (1). The report categorized the participant as low, moderate, or high fracture risk. The FRAX report (Appendix 2) used participants’ femoral neck BMD and clinical risk factors incorporated by the FRAX tool; Canadian population data were used (35). The report indicated which clinical risk factor(s) were present and the percent probability of a major osteoporotic fracture over the next 10 yr. The FRAX report highlighted the fracture risk category and included statements regarding treatment recommendations as per clinical guidelines (1). Before the study, feedback received from 8 nonparticipating FPs regarding the layout and content of the FRAX was used to inform the revised FRAX report used in the study. Study participants did not receive a copy of their fracture risk report. Three months after reports were sent to FPs, participants were called and asked a series of questions regarding follow-up by their FP including whether the participant had been contacted by their FP, whether the FP discussed their results with them, and whether they were recommended for treatment. Participants who could not be reached at 3 months received a second follow-up phone call 1 month later.

To determine if FPs follow-up (i.e., treatment recommendation or not) was appropriate, all reports were read by an osteoporosis specialist (J.D.A.) who was considered the reference for comparison. Only information on the report was available to the specialist, who was blinded to treatment decisions made by FPs.

To confirm participants’ responses regarding follow-up care, we attempted to contact FPs. A questionnaire was sent to each FP to confirm participants’ responses regarding follow-up care and to corroborate information regarding treatment. An additional questionnaire was distributed to FPs allocated to the FRAX report group to survey their understanding and preference for the FRAX report over the standard BMD report. FPs who responded received a gift card in appreciation of their time. The study was approved by the Research Ethics Boards at McMaster University Faculty of Health Sciences/ Hamilton Health Sciences and the University of Western Ontario.

### Statistical Analyses

Descriptive statistics were conducted to characterize the study population. Kappa statistics and 95% confidence intervals (CIs) were calculated to determine agreement between FPs’ treatment decisions compared with those of the osteoporosis specialist. A Fisher’s exact test was used to compare the differences between the treatment rates in the 2 groups and whether there was a significant difference in the number of FPs’ discussion with patients between the standard BMD report group and the FRAX group. Participants’ responses regarding the follow-up they received from their FPs are summarized as are the questionnaire responses from FPs regarding their feedback on the FRAX reports.

## Results

Participants’ demographic information (mean [SD] age 65 ± 9.5 yr, 82% women) is shown (Table 1). Randomization resulted in the allocation of 29 FPs to receive a standard BMD report and 31 FPs to receive an FRAX report. In the standard BMD report group, 20 participants were at moderate fracture risk whereas 9 were at high risk. In the FRAX report group, 11 were at low fracture risk, 16 at moderate risk, and 4 at high risk.

In following up with study participants in the standard BMD report group, 8 of 29 (27.6%) reported being contacted by their FPs (5 moderate, 3 high) whereas 14 of 29 (45.6%; 9 moderate risk, 5 high risk) reported discussing their results with their FP. In the FRAX report group, 14 of 31 (45.2%) participants were contacted by their FP (5 low, 6 moderate, 3 high) whereas 20 of 31 (64.5%) participants (7 low, 10 moderate, 3 high) reported discussing their results with their FP. Although a 19% greater rate of discussion with FPs that occurred in the FRAX group may be clinically relevant, this difference was not statistically significant ( *p* = 0.29). Five participants in each group reported being recommended for pharmacologic therapy (Table 2). One of the 2 additional high-risk participants in the FRAX group was not followed-up by her FP even 4 months after BMD testing, and one was not treated despite discussing her results with her FP. In the standard BMD report group, 15 of 29 (51.7%) FPs were successfully contacted to corroborate their patients’ responses compared with 20 of 31 (64.5%) in the FRAX group. In all cases, FPs’ responses were consistent with participants regarding follow-up care. In comparing treatment decisions made by the FPs with those of an independent, blinded osteoporosis specialist, FPs recommended treatment in 5 patients allocated to the standard BMD group, compared with 8 patients recommended by the specialist. Only 3 of these participants were recommended for treatment by both the physicians yielding an overall kappa statistic of agreement of 0.32 (95% CI 0.00–0.70). In the FRAX group, FPs recommended treatment for 5 patients compared with 9 by the specialist. All 5 patients treated by the FPs also were treated by the specialist, yielding an overall kappa statistic of 0.64 (95% CI 0.33–0.95). No participants at low risk were recommended for treatment by either the physician.

**Fig. 1:**
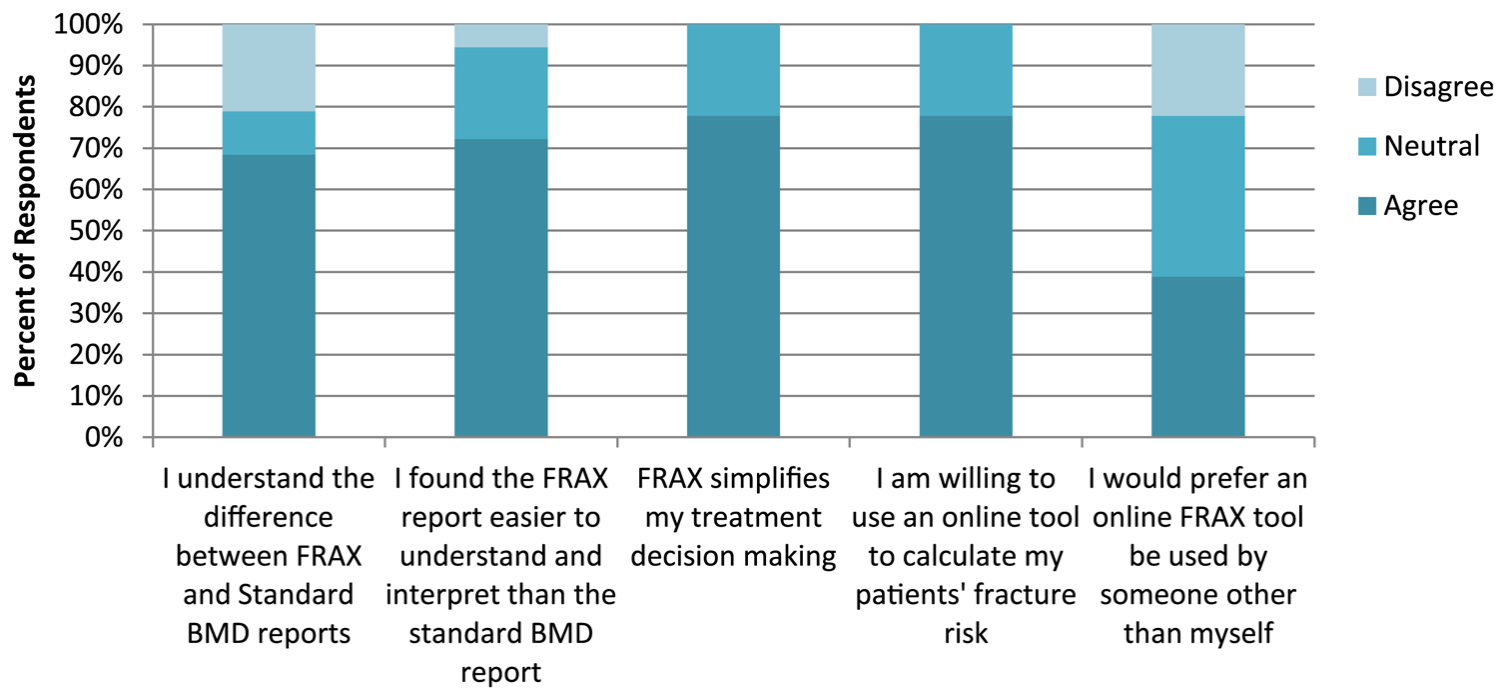
Summary of family physicians’ responses to questions regarding their understanding and preference for FRAX reports compared with standard BMD reports. ‘‘Agree’’ responses are a composite of those who responded to strongly agree, moderately agree, and mildly agree. Responses of strongly, moderately and mildly disagree are represented by ‘‘Disagree.’’ BMD, bone mineral disease; FRAX, fracture risk assessment tool.

Nineteen of 31 FPs (61.2%) who received FRAX reports responded to questionnaires regarding their understanding and preference for the FRAX reports vs the standard BMD reports. Answers were given on a 7-point Likert scale ranging from ‘‘strongly disagree’’ to ‘‘strongly agree.’’ Feedback regarding these reports was generally positive (Fig. 1).

## Discussion

This study evaluated the outcomes of care provided by FPs to their patients after routine treatment for a wrist fracture. There was a greater rate of treatment (44% vs 50%) in high-risk participants whose FPs received the FRAX report. Agreement between the osteoporosis specialist and the FPs’ treatment recommendations also was higher for the FRAX report. Compared with standard CAROC-based BMD reports FPs routinely received from the center, which made no directive treatment recommendations, FPs who received the FRAX report understood the report, found it easier to explain to their patients, and agreed that it simplified their treatment decision.

Currently, Canadian guidelines (1) recommend that individuals at high fracture risk be treated; those at moderate risk (10%–20%) should at least be considered for pharmaco-therapy if other risk factors coexist, and those at low fracture risk (<10%) should not be treated. These categories provide a pathway to evaluate treatment decisions following bone densitometry. Approximately 3 months after enrollment, 28% of participants in the standard BMD group had been contacted by their FPs, and 46% had discussed their results with their FPs. The difference in these figures might result from patients visiting their FPs as a follow-up to their fracture or regarding a different concern, yet discussed the results of their BMD report. However, only 3 of 9 participants at high fracture risk, when the standard BMD report was used, discussed their results with their FPs.

For participants allocated to the FRAX report group, outcomes were more consistent. Here, 45% of participants were contacted by their FPs and 65% discussed their results. Some discussion of the FRAX report took place across all levels of fracture risk; 64% at low risk, 38% at moderate risk, and 75% at high risk discussed their results with their FP. Although differences in frequencies of discussions with patients between groups were not statistically significant, the FRAX report appears to have triggered a more clinically consistent response, which is encouraging and worth exploring in a larger prospective study.

The FRAX tool was developed as a means of estimating absolute fracture risk thereby simplifying treatment decisions by establishing country-specific fracture risk treatment thresholds. Overall, there was a clinically greater degree of agreement of treatment recommendations with the specialist with FPs who received the FRAX report compared with the standard BMD report. Given all participants had a fragility fracture, no participants in the standard BMD group were at low fracture risk. Therefore, all participants would be at least considered for treatment by the FP. Only 4 of 9 participants (44%) allocated to the standard BMD report were recommended for treatment by their FPs, which is concerning given that guidelines recommend individuals at high fracture risk be treated. In the FRAX group, none of the 11 participants at low fracture risk were treated. Comparatively, although only half of the high-risk patients assessed by FRAX were treated pharmacologically (2/4), results were at least discussed by a third patient. Small numbers of individuals in the high-fracture risk groups make it difficult to conclude that high fracture risk patients were treated more appropriately by FPs who received the FRAX report.

Treatment recommendations for patients at moderate fracture risk remain problematic (36) because either treatment or no treatment might be appropriate depending on individual circumstances. The specialist was less likely to recommend treatment for patients at the lower end of ‘‘moderate risk’’ (e.g., 11%–13%) than the higher end (e.g., 17%–19%). Unlike the CAROC tool, which provides categorical risk, the FRAX report facilitates this approach by providing a point estimate of risk. Only 1 of 20 participants at moderate risk in the standard BMD report group was recommended for treatment by her FP. The specialist did not recommend treatment in this participant. Of the 16 patients at moderate risk by the FRAX report, the specialist would have treated 5. The FPs discussed the FRAX reports with all 5 of these participants, and 3 were treated.

No other studies have prospectively assessed the effects of an FRAX report on treatment behavior of physicians, although 3 retrospective studies have been conducted (32,36,37). Two studies compared physicians’ prescribing behaviors between a period before the introduction of absolute fracture risk reporting compared with afterward. In one study, conducted only in osteopenic individuals (>83% male), investigators concluded that the inclusion of FRAX in a bone density report had no effect on physicans’ prescribing behavior (36). However, this study included a predominantly male population, and the lack of knowledge about FRAX may have led physicians not to consider this information in their decision making. In contrast, Leslie et al (32) concluded that the transition from a T-score based fracture risk report to a report based on absolute fracture risk resulted in appropriate, guideline-based changes in pharmacological treatment. This was based on fewer lower- and moderate-risk patients being treated with the absolute fracture risk reporting system. These observations are consistent with our results, in which 11 participants at low fracture risk in the FRAX group would not be treated. The third study investigated agreement between rheumatologists on whether to treat a patient after reading a T-score based report compared with an absolute fracture risk report. A greater agreement was observed after reading absolute fracture risk reports, resulting in improved consistency and perhaps efficiency in treating patients (37).

Feedback from FPs regarding the FRAX report was positive. Of all respondents (62%), FRAX reports were well understood and aided in simplifying treatment decision making (Fig. 1). These results are consistent with previous studies (37,38). However, it is not clear whether the FRAX report itself or the accompanying evidence-based guidelines on treatment according to fracture risk category influenced FP treatment decisions.

This study suffers from several limitations. There were too few participants at moderate or high fracture risk to determine whether the FRAX report group had a significantly improved treatment rate. Although the FRAX report contained a point estimate of fracture risk, emphasized risk categorization, and structured evidence-based treatment recommendations in accordance with the risk category, the routinely used CAROC-based standard report provided only the risk category without treatment recommendation. Thus, it is not possible to isolate the particular components of the report that influenced treatment outcomes. Thus, we cannot speculate whether an optimized CAROC-based report would have affected treatment outcomes. However, as previously shown (32), our results suggest that the CAROC-based report leads to systematic overtreatment of patients with wrist fractures. We did not evaluate the potential additional impact that giving copies of the fracture risk report to patients may have had on treatment recommendations by the FP. In addition, only 58% of FPs returned requests to corroborate their study participants’ responses. Although there was complete agreement among respondents, we cannot generalize this to the remaining cases.

In summary, a greater proportion of FPs who received a FRAX report contacted their patients and discussed the test implications compared with FPs who received the standard BMD report. There was greater agreement in treatment outcomes between the osteoporosis specialist and FPs who received the FRAX report than the standard BMD report. Despite few participants at high fracture risk, there was a greater treatment rate of participants whose FPs received the FRAX report. If these results can be repeated in a larger study population, the use of a more appropriately structured BMD report based on the FRAX tool coupled with simple evidence-based treatment recommendations has the potential to reduce the existing treatment care gap following a wrist fracture.

## Figures and Tables

**Table 1:** Descriptive Statistics for the Study Population

Variable	Entire group (n = 60) Mean (SD)/n (%)	Standard BMD (n = 29) Mean (SD)/n (%)	FRAX (n = 31) Mean (SD)/n (%)
Age, yr	65.0 (9.5)	65.3 (9.4)	64.7 (9.7)
BMI, kg/m^2^	27.0 (5.4)	27.3 (5.7)	26.8 (5.1)
Femoral neck BMD, g/cm^2^	0.86 (0.11)	0.87 (0.10)	0.85 (0.12)
Lumbar spine BMD, g/cm^2^	1.14 (0.20)	1.13 (0.15)	1.14 (0.24)
Femoral neck T-score	−1.12 (0.91)	−1.03 (0.91)	−1.20 (0.93)
Lumbar spine T-score	−0.56 (1.66)	−0.61 (1.30)	−0.51 (1.96)
Female	49 (81.7)	24 (86.2)	24 (77.4)
Current smoker	7 (11.7)	2 (6.9)	5 (16.1)
Family history of osteoporosis	18 (30.0)	7/28 (25)	11/30 (36.7)
Heart disease	5 (8.3)	2/29 (6.9)	3/31 (9.7)
High blood pressure	21 (35.0)	11/29 (37.9)	10/31 (32.3)
Lung disease	2 (3.3)	1/29 (3.4)	1/31 (3.2)
Diabetes	3 (5.0)	1/29 (3.4)	2/31 (6.5)
Ulcer/stomach disease	5 (8.3)	2/29 (6.9)	3/31 (9.7)
Anemia/blood disease	2 (3.3)	1/29 (3.4)	1/31 (3.2)
Cancer	6 (10.0)	1/29 (3.4)	5/30 (16.7)
Depression	10 (16.7)	4/29 (13.8)	6/31 (19.4)
OA/degenerative arthritis	15 (25.0)	7/29 (24.1)	8/31 (25.8)
Back pain	21 (35.0)	9/29 (31.0)	12/30 (40.0)
Rheumatoid arthritis	3 (5.0)	2/28 (7.1)	1/31 (3.2)

*Abbr:* BMD, bone mineral density; FRAX, fracture risk assessment tool; OA, osteoarthritis.

**Table 2:** The Number and Proportion (%) of Participants Recommended for Pharmacologic Therapy by Their Family Physicians and the Osteoporosis Specialist

	Number treated (%) by family physician	Number treated (%) by osteoporosis specialist
Standard BMD		
Moderate	1/20 (5.0)	0/20 (0)
High	4/9 (44.4)	8/9 (88.9)
FRAX		
Low	0/11 (0)	0/11 (0)
Moderate	3/16 (18.9)	5/16 (31.3)
High	2/4 (50.0)	4/4 (100.0)

*Abbr:* BMD, bone mineral disease; FRAX, fracture risk assessment tool.
